# [Retracted] The AMPK‑mTOR axis requires increased MALAT1 expression for promoting granulosa cell proliferation in endometriosis

**DOI:** 10.3892/etm.2024.12528

**Published:** 2024-04-02

**Authors:** Xuejie Liu, Ping Zhang, Yanmin Li, Na Zhao, Haiyan Han

Exp Ther Med 21:21, 2021; DOI: 10.3892/etm.2020.9453

Following the publication of this paper, it was drawn to the Editors’ attention by a concerned reader that the EdU staining assay data shown in Fig. 2C and the immunofluorescence assay data shown in Fig. 4A and 6E were strikingly similar to data appearing in different form in other articles written by different authors at different research institutes that had already been published elsewhere prior to the submission of this paper to *Experimental and Therapeutic Medicine* (one of which has subsequently been retracted). In view of the fact that the abovementioned data had already apparently been published previously, the Editor of *Experimental and Therapeutic Medicine* has decided that this paper should be retracted from the Journal. After having been in contact with the authors, they accepted the decision to retract the paper. The Editor apologizes to the readership for any inconvenience caused.

